# Managing the cancer backlog: a national population-based study of patient mobility, waiting times and ‘spare capacity’ for cancer surgery

**DOI:** 10.1016/j.lanepe.2023.100642

**Published:** 2023-05-03

**Authors:** Ajay Aggarwal, Lu Han, Richard Sullivan, Kate Haire, Vijay Sangar, Jan van der Meulen

**Affiliations:** a Department of Health Services Research and Policy, London School of Hygiene and Tropical Medicine, London, UK; b Department of Oncology, Guy's and St Thomas' NHS Foundation Trust, London, UK; c Institute of Cancer Policy, King's College London, London, UK; d South East London Cancer Alliance, UK; e The Christie NHS Trust and Manchester University NHS Foundation Trust, Manchester, UK; f Manchester University, UK

**Keywords:** Waiting times, Cancer surgery, Patient mobility, Capacity, Treatment backlog

## Abstract

**Background:**

Waiting times for cancer treatments continue to increase in many countries. In this study we estimated potential ‘spare surgical capacity’ in the English NHS and identified regions more likely to have spare capacity based on patterns of patient mobility (the extent to which patients receive surgery at hospitals other than their nearest).

**Methods:**

We identified patients who had an elective breast or colorectal cancer surgical resection between January 2016 and December 2018. We estimated each hospital's ‘maximum surgical capacity’ as the maximum 6-month moving average of its surgical volume. ‘Spare surgical capacity’ was estimated as the difference between maximum surgical capacity and observed surgical volume. We assessed the association between spare surgical capacity and whether a hospital performed more or fewer procedures than expected due to patient mobility as well as the association between spare surgical capacity and whether or not waiting times targets for treatment were likely to be met.

**Findings:**

100,585 and 49,445 patients underwent breast and colorectal cancer surgery respectively. 67 of 166 hospitals (40.4%) providing breast cancer surgery and 82 of 163 hospitals (50.3%) providing colorectal cancer surgery used less than 80% of their maximum surgical capacity. Hospitals with a ‘net loss’ of patients to hospitals further away had more potential spare capacity than hospitals with a ‘net gain’ of patients (p < 0.001 for breast and p = 0.01 for colorectal cancer). At the national level, we projected an annual potential spare capacity of 8389 breast cancer and 4262 colorectal cancer surgical procedures, approximately 25% of the volumes actually performed.

**Interpretation:**

Spare surgical capacity potentially exists in the present configuration of hospitals providing cancer surgery and requires regional allocation for efficient utilisation.

**Funding:**

10.13039/501100000272National Institute for Health Research.

**Research in context**

**Evidence before this study**

There is increasing demand for cancer services in many countries. Whilst investments in creating additional workforce and expanding capacity are being considered, a short-term public health solution is urgently required to address current delays in treatment and reduce the expected number of avoidable cancer deaths. We searched PubMed for full-text articles, published between 1st January 1990, and 1st December 2022, to assess the different approaches that have been used to estimate ‘spare cancer treatment capacity’. The search was restricted to English language publications. Search terms included (‘capacity’ OR ‘waiting times’) AND (‘cancer’). The studies we identified assessed how much surgical capacity is required to keep waiting times for cancer surgery within accepted standards, what capacity is required to meet increasing demand, and what internal hospital management processes have been implemented to reducing waiting times. No studies were identified that estimated potential spare treatment capacity on a national level.

**Added value of this study**

Our study considered ‘spare surgical capacity’ as well as patient mobility patterns and waiting times for cancer surgery, derived from linked routinely collected national datasets of patients undergoing breast and colorectal cancer resections in the English NHS between January 2016 and December 2018. We identified potential ‘spare surgical capacity’ based on 6-month moving averages of the hospitals' surgical volumes. We found that hospitals with a ‘net loss’ of patients (i.e., hospitals that perform fewer procedures than expected if all patients would be treated at the hospital nearest to them) had potentially more spare capacity than hospitals with a ‘net gain’ of patients. At the national level, we projected a potential annual spare surgical capacity of about 8000 surgical procedures for breast cancer and about 4000 surgical procedures for colorectal cancer which, if fully used, could increase the annual number of patients having breast cancer or colorectal cancer surgery by 25%. This study demonstrates the importance of systems-wide analysis of surgical capacity for planning cancer care.

**Implications of all the available evidence**

Our study highlights the importance of considering patient mobility as a factor influencing cancer available potential spare capacity in hospitals providing cancer surgery. It is unlikely that these findings are unique to the NHS and apply to many other countries with state-funded healthcare of universal health insurance coverage that allow patients to choose where they have their cancer treatment. Our findings demonstrate the need for regional coordination to reduce treatment backlogs and optimise the use of cancer surgery capacity through an assessment of patient referral patterns and existing hospital level workload. A more radical consideration is that existing patterns of patient mobility for cancer surgery forms the basis for centralisation or specialisation of services.

## Introduction

The backlog in the treatment of cancer patients is one of the most significant current health policy issues in the United Kingdom (UK) and many other countries.^1^ The delays in treatment pathways, in part due to the COVID-19 pandemic is expected to result in significant numbers of avoidable deaths over the next five years.^2^, ^3^, ^4^, ^5^, ^6^, ^7^

A recent report published by the National Audit Office, an independent body in the UK responsible for auditing central government departments, highlighted the overall inadequacy of the current diagnostic and treatment capacity in the English NHS. Factors such as staffing, availability of beds including critical care, specialist referral pathways for complex procedures, and the volume of cancer and non-cancer surgical procedures performed are all relevant. The report concluded that this lack of capacity in itself makes it unlikely that initiatives to reduce long waits for cancer care services will be successful, even by 2025.^8^^,^^9^

Despite this, in March 2022, the English Secretary of State for Health and Social Care introduced a flagship policy ‘My Planned Care’ to clear the elective treatment backlog. A central tenet underpinning the policy is that spare treatment capacity is likely to exist in the National Health Service (NHS), with newly diagnosed patients having the opportunity to move to a NHS hospital outside their local area so that they can have their treatment in hospitals with shorter waiting times.^10^ This policy was meant to formally start in December 2022 providing information on average treatment waiting times for a particular sub-speciality. It is initially available to patients with very long waits for elective treatment before being extended to all patients.

However, a policy that simply relies on patient mobility to tackle waiting lists needs a critical review for several reasons.^11^ First, there are many characteristics other than the length of the cancer waiting times that make a hospital attractive to patients. For example, the presence of more advanced treatment modalities, its reputation in local and national media, and having better patient outcomes.^12^^,^^13^ This means that patients may even prefer to wait longer for treatment in a particular hospital if—for whatever reason—they expect that this hospital provides better cancer care.

Second, we have shown in previous studies that up to 30% of patients with bowel and prostate cancer bypass their nearest treating hospital, which results in some hospitals having a ‘net gain’ of patients (hospitals that perform more procedures than expected if all patients would be treated at the hospital nearest to them) and other hospitals having a ‘net loss’.^14^, ^15^, ^16^ A systematic review appraising international studies exploring the extent of patient mobility for a wide range of elective secondary care services, found that 23–77% of patients seek care at alternative providers and that the influx of patients from out of areas may result in lengthening waiting lists.^12^

Numerous studies have sought to understand how the flow of patients between catchment areas can provide a competitive incentive for quality improvement within health care markets.^17^ In addition, a population-based study in prostate cancer demonstrated that centres with a net loss of patients were more likely to close their prostate cancer surgical service compared to those with a net gain.^18^

What we do not know from the current literature is the implications of patient mobility on hospital capacity, and whether those hospitals that have a net loss as a result of patient mobility potentially have additional capacity for treatment. To answer if this association exists, we carried out a national population-based study analysing patient mobility patterns, waiting times, and potential hospital capacity for breast and colorectal cancer surgery in the English NHS between 2016 and 2018, using existing linked national cancer registry and administrative hospital data.

First, we compared cancer waiting times between hospitals with a net gain and hospitals with a net loss of patients. Second, we estimated the hospitals' potential ‘spare surgical capacity’ and ‘actual surgical capacity usage’, comparing the hospitals' average surgical volume with their maximum capacity (see Methods). Third, we assessed the differences in the hospitals' actual surgical capacity usage between hospitals with a net gain and hospitals with a net loss of patients. Finally, we estimated the potential spare surgical capacity for breast and colorectal cancer surgery at the national level within Cancer Alliances, the organisational structures in the English NHS that coordinate diagnosis, treatment and care for cancer patients in 21 regions.^19^ Based on the results of these analyses, we developed recommendations to reduce the backlog in the treatment of newly diagnosed cancer patients.

## Methods

### Data sources and study population

We obtained patient-level data for all patients with breast and colorectal cancer who underwent major cancer resection in an English NHS hospital between 1st January 2016 and 31st December 2018. Patients undergoing surgery had been diagnosed between 1st January 2013 and 31st December 2018.

Data provided by the English Cancer Registry included demographic characteristics, date of diagnosis, cancer stage^20^ and the number of comorbidities.^21^ The linked Hospital Episode Statistics (HES), the administrative database of all care episodes in English NHS hospitals, provided information on the treating NHS hospital site, the date of admission for the major cancer surgery, the mode of admission (i.e., elective or urgent), and the type of resection. In addition, it provided socioeconomic deprivation expressed in terms of quintiles of the national distribution of the Index of Multiple Deprivation (IMD) of the patient's residential location represented by for 32,844 Lower Super Output Areas (LSOA) which are geographical footprints representing up to 1500 people and 650 households.^22^ Diagnoses were coded using the International Classification of Disease, 10th Revision (ICD-10)^23^ and procedure information was coded according to the Office of Population Censuses and Surveys Classification of Surgical Operations and Procedures, 4th Revision (OPCS-4).^24^

Breast cancer patients were identified in the Cancer Registry data using the ICD-10 code C50. Patients with these breast cancer codes were included if their sex was recorded as ‘female’ and if there was no other cancer diagnosis one month before and one month after the breast cancer diagnosis. For patients with multiple diagnoses of breast cancer in the Cancer Registry data, we used information on the earliest diagnosis record. Patients undergoing breast conserving surgery were identified with the following OPCS-4 codes: B281, B282, B283, B285, B287, B288, B289, B411, B412 and B419 in the linked HES records. Patients undergoing mastectomies were identified with OPCS-4 code: B27. We then identified the 166 NHS hospitals that provided these procedures in the study period between 1st January 2016 and 31st December 2018.

A similar process was followed to identify colorectal cancer patients. Patients diagnosed with colorectal cancer were identified in the Cancer Registry data using ICD-10 codes C18-C20. Patients undergoing a major colorectal cancer resection were identified with the following OPCS-4 codes: H04, H05, H06, H07, H08, H09, H10, H11, H29, H33, H411, H414, X141, X142, X143, X148 and X149. All hospitals offering surgery for colorectal cancer treat both colon and rectal cancers. We identified 163 NHS Hospitals that provided these procedures in the study period between 1st January 2016 and 31st December 2018.

### Patient mobility

The patients' residential location was represented by the population-weighted centroids of the LSOA.^22^ A geographic information system (ESRI ARC GIS) was used to determine the average daytime travel times by private car between the patients’ residential location and each of the NHS hospitals providing breast cancer or colorectal cancer surgery, which were ranked to identify the nearest hospital for each patient.

For each hospital, we distinguished three patient groups. First, we defined the ‘core patients’ as those patients for whom that hospital was the nearest and who had their surgery at that hospital. Second, we defined the ‘leavers’ as those patients for whom that hospital was the nearest but who had their treatment at another hospital further away. Third, we defined the ‘arrivers’ as those patients who had their surgery at that hospital but for whom another was the nearest. The ‘net gain’ or ‘net loss’ of patients for each hospital was calculated as the difference between the numbers of arrivers and the number of leavers.^18^ We used the conditional method for testing a difference between two Poisson means to compare the number of arrivers and leavers or, in other words, whether the net loss or net gain of patients was statistically significantly different from zero.^25^^,^^26^ The net gains or net losses were averaged over the 3-year study period to calculate the annual net gains and losses. We looked at key differences in the attributes of hospitals with a net gain or net loss of patients as previously described.^16^

### ‘Spare capacity’ estimation

**Fig. 1:**
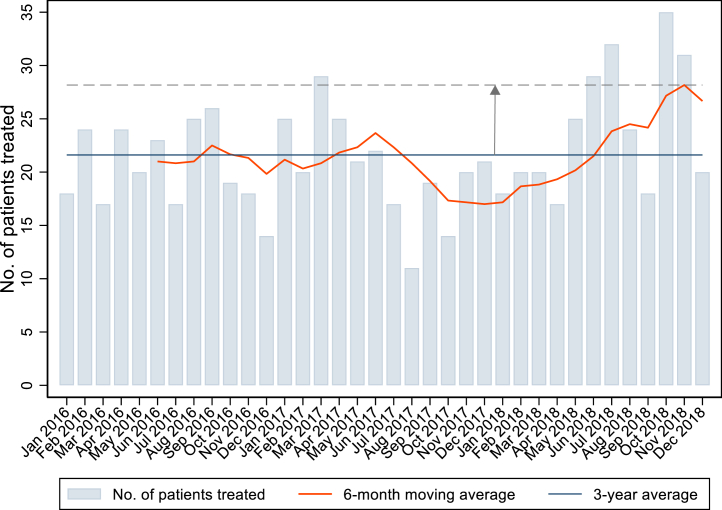
**Monthly breast cancer surgery procedure volumes at a selected NHS centre between Jan 2016 and Dec 2018, plotted with the 3-year mean and 6-month moving average**. Notes: See methods section for estimation of the 6-month moving average. The dashed line represents the maximum monthly surgical capacity based on the 6-month moving average. Spare surgical capacity was estimated as the difference between the maximum surgical capacity based on the 6-month moving average and the average monthly surgical volume over the 3-year period.

Patient-level hospital admissions were aggregated at the hospital level to obtain monthly volumes of breast cancer resections (including breast conserving surgery and mastectomies) and monthly volume of major bowel resections during the study period between 1st January 2016 and 31st December 2018. Within this 3-year period, an estimate of a hospital's potential ‘spare capacity’ was estimated separately for breast cancer surgery and for bowel cancer surgery following a stepwise procedure as illustrated in Fig. 1.

First, we estimated the ‘maximum surgical capacity’ of a hospital as the maximum of the 6-month moving averages of the monthly surgical volumes (dashed horizontal line in Fig. 1). We used the moving average over a 6-month period to reduce the impact of the inherent ‘volatility’ in surgical volumes over time. We chose a 6-month period because we assumed that an average monthly volume over this period is likely to provide a realistic estimate of a hospital's maximum surgical capacity in a steady state while also allowing for systematic fluctuations in surgical capacity over time.^27^

Second, we estimated a hospital's ‘average monthly surgical volume’ over the entire 36 months of the study period (solid horizontal line in Fig. 1). Third, we determined a hospital's potential ‘spare capacity’ as the difference between a hospital's maximum surgical capacity and average monthly surgical volume (vertical black arrow in Fig. 1):

sparesurgicalcapacity=maximumsurgicalcapacity–averagemonthlysurgicalvolume

We then estimated the ‘actual surgical capacity usage’ as the ratio of the average surgical volume and the maximum surgical capacity expressed as a percentage:

$$actual\;surgical\;capacity\;usage=\;\;\frac{average\;monthly\;surgical\;volume}{maximum\;surgical\;capacity}\;\times \;100\%$$

The same stepwise process was repeated to calculate spare capacity and the actual surgical capacity usage at regional levels, using geographic areas covered by the 21 NHS Cancer Alliances.

### Cancer waiting time target

The current target in England is to start treatment within 31 days from the decision to treat date.^28^ Using publicly accessible NHS data, we aggregated monthly patient volumes across three years from 2016 to 2018 for each hospital and grouped the hospitals into two categories according to whether the percentage of patients meeting this cancer waiting time target was above or below 94% (which is the national waiting time performance target).

### Statistical analysis

We used contingency tables to explore associations between the hospital-level characteristics. These associations were statistically tested using Pearson chi-squared tests. Fisher's exact tests were used if the expected number of hospitals in any cell of the contingency table was lower than five. All data analyses were conducted in Stata 17.

### Role of the funding source

The funder of the study had no role in study design, data collection, data analysis, data interpretation, or writing of the report.

## Results

### Patient characteristics

We identified 106,125 elective breast cancer procedures between 1st January 2016 and 31st December 2018 in 100,585 patients with breast cancer, including 70,544 breast conserving procedures and 35,581 mastectomies (Appendix Fig. S1). We identified 49,993 elective major bowel cancer resections in 49,445 patients with colorectal cancer, including 35,294 colon cancer resections and 14,699 rectal cancer resections (Appendix Fig. S2).

The mean age of the breast cancer patients was 61.0 years, 86.8% had Stage I or II disease, and 10.9% had one or more comorbidities (Table 1). Patients who had their surgery at their nearest hospital were comparatively older (mean age 61.4 years) compared to those who were treated at an alternative more distant hospital (mean age 60.3 years). The differences in socioeconomic status was minimal but a larger proportion of patients moving to alternative hospitals tended to live in rural areas or were from London (Appendix Table S1).

**Table 1:** Patient characteristics.

	Breast conserving and mastectomy surgeries (n = 106,125)		Major bowel resections (n = 49,993)	
	n	%	n	%
**No. of patients**	100,585	100	49,445	100
**Age (years), mean (SD)**	61.02 (13.0)		68.57 (11.79)	
**Sex**				
Male			28,388	57.4
Female	100,585	100	21,057	42.6
**Ethnicity**				
White	88,309	87.8	45,062	91.1
Asian	3568	3.6	1041	2.1
Black	2082	2.1	710	1.4
Mixed	607	0.6	157	0.3
Other	1982	2.0	681	1.4
Not known/missing	4037	4.0	1794	3.6
**Cancer stage**				
Stage 1	46,565	46.3	10,043	20.3
Stage 2	40,753	40.5	16,550	33.5
Stage 3	8937	8.9	17,764	35.9
Stage 4	1111	1.1	3794	7.7
Not known/missing	3219	3.2	1294	2.6
**Number of comorbidities according to RCS Charlson Score**				
0	89,623	89.1	40,887	82.7
1	6759	6.7	4486	9.1
2+	4203	4.2	4072	8.2
**Index of multiple deprivation (IMD)**				
1st quintile (least deprived)	22,725	22.6	11,439	23.1
2nd quintile	23,224	23.1	11,734	23.7
3rd quintile	21,078	21.0	10,466	21.2
4th quintile	18,143	18.0	8554	17.3
5th quintile (most deprived)	15,415	15.3	7252	14.7

The mean age of the colorectal cancer patients was 68.6 years, 57.4% were men, and 53.8% had Stage I or II disease, and 17.3% had one or more comorbidities (Table 1). Patients who had their surgery at their nearest hospital were comparatively older (mean age 69.1 years) and from more deprived areas (32.8% IMD 4 or 5) compared to those who were treated at an alternative more distant hospital (mean age 67.3 years and 30% IMD 4 or 5). A larger proportion of patients moving to other hospitals tended to live in rural areas or London (Appendix Table S1).

### Hospitals with net gain and net loss of patients

**Fig. 2:**
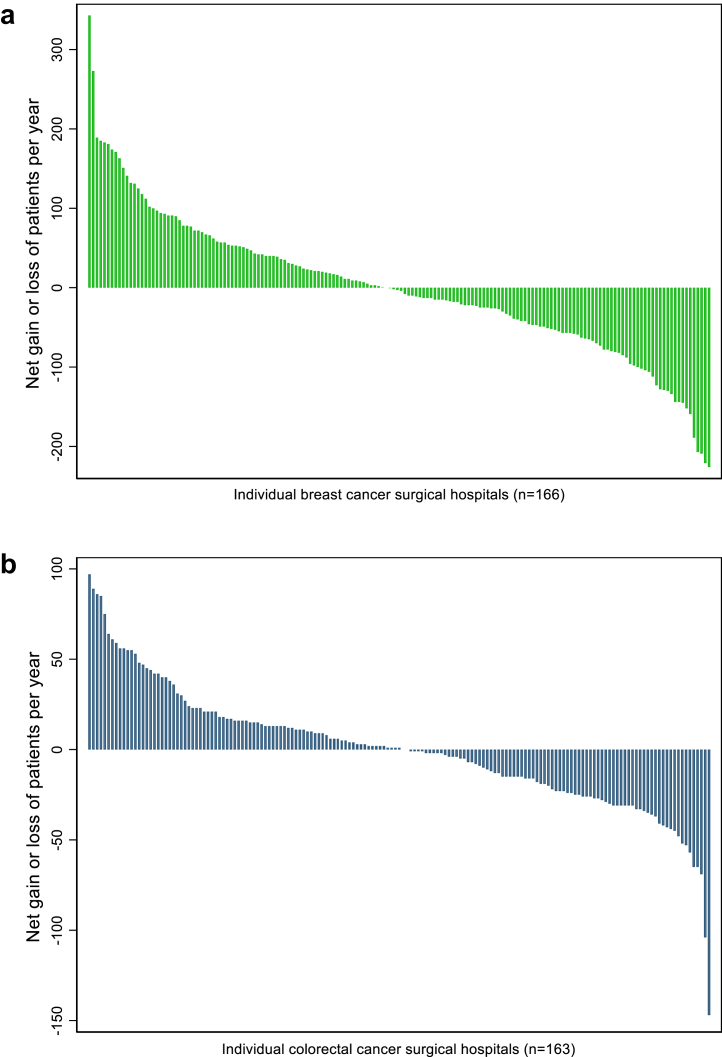
**a) Net gains and losses of patients due to patient mobility for each hospital providing breast cancer surgery between January 2016 and December 2018. b) Net gains and losses of patients due to patient mobility for each hospital providing colorectal cancer surgery between January 2016 and December 2018**.

Fig. 2a shows the net gain or net loss of patients per year for each of the 166 hospitals performing breast cancer surgery as a result of patient mobility. 72 hospitals (43.4%) had a statistically significant annual net gain of patients, and 81 centres (48.8%) had an annual net loss. We observed that 18 hospitals (10.8%) had an annual net gain of 100 or more procedures and 19 hospitals (11.4%) had an annual net loss of 100 procedures or more. The average number of procedures performed each year by hospitals with a net gain was 285 compared to 147 for hospitals with a net loss. In addition, hospitals with a net gain were more likely to be centres that offered breast reconstruction surgery and to be comprehensive cancer centres although the latter was not statistically significant (Appendix Table S2).

Fig. 2b shows a similar pattern for the 163 hospitals performing colorectal cancer surgery. 63 hospitals (38.7%) had a statistically significant annual net gain and 66 hospitals (40.5%) had annual net loss. 24 hospitals (14.7%) had an annual gain of 30 procedures or more procedures and 20 hospitals (12.3%) an annual net loss of 30 procedures or more. The average number of procedures performed each year by hospitals with a net gain was 118 compared to 84 for hospitals with a net loss. In addition, hospitals with a net gain were more likely to be specialist colorectal units and performed a larger volume of highly complex procedures such as pelvic exenterations compared to hospitals with a net loss. However, this association was not statistically significant (Appendix Table S3).

Of the 125 hospitals performing both breast cancer and colorectal cancer surgery, we found that hospitals with a net gain of patients (and hence performed more surgical procedures than expected) for breast cancer surgery were also more likely to have a net gain of patients for colorectal cancer surgery (p < 0.001; Appendix Table S4). For example, of the 52 hospitals performing breast cancer surgery with a net gain of patients, 35 hospitals (67.3%) also had a net gain for colorectal cancer. Similarly, of the 63 hospitals performing breast cancer surgery with a net loss of patients, 39 (61.9%) also had a net loss for colorectal cancer. Appendix Fig. S3a and b demonstrate the variation in bypass rates for breast cancer and colorectal cancer surgery respectively across the NHS Cancer Alliances.

### Cancer waiting times in hospitals with a net gain or net loss of patients

We evaluated the association between a hospital having a gain or net loss of patients and whether it met the 31-day cancer waiting time target (Appendix Table S5). Fewer hospitals with a net gain had met the waiting list target, however this association was not statistically significant. For breast cancer surgery, 21 of the 72 hospitals with a net gain of patients (29.2%) did not meet the waiting time target compared to 12 of the 81 hospitals with a net loss (14.8%; p = 0.09). Corresponding results for colorectal cancer show that 17 of 63 hospitals with a net gain of patients (27.0%) did not meet the waiting time target and 12 of the 66 hospitals with a net loss (18.2%; p = 0.31).

### Actual surgical capacity usage

Using our measure for actual surgical capacity usage, we found that 67 of the 166 hospitals (40.4%) providing breast cancer surgery used less than 80% of their maximum capacity and 21 hospitals (12.7%) used less than 70% (Appendix Table S6). Similar results for colorectal surgery demonstrate that 82 of the 163 hospitals (50.3%) used less than 80% of their maximum capacity and 19 hospitals (11.7%) less than 70% of their maximum capacity. Only three hospitals were found to be using more than 90% or above of their maximum capacity for both cancer types.

### Actual surgical capacity usage in hospitals with a net gain or net loss of patients

For both cancers, hospitals with a net gain of patients were more likely to use 80% or more of their maximum surgical capacity than hospitals with a net loss (Table 2). For breast cancer surgery, 54 of the 72 hospitals (75.0%) with a net gain of patients used 80% or more of their maximum surgical capacity, compared to 34 of the 81 hospitals (42.0%) with a net loss (p < 0.001). This association was present and statistically significant when considering breast conserving surgery and mastectomy separately. For colorectal cancer, 37 of the 63 hospitals (58.7%) with a net gain of patients used 80% of their maximum surgical capacity, compared to 22 of 66 hospitals (33.3%) with a net loss (p = 0.01).

**Table 2:** Number of hospitals performing breast cancer surgery or colorectal cancer surgery with a net gain or net loss of patients according to their actual surgical capacity usage.

	Actual surgical capacity usage^a^ (% of maximum surgical capacity)				
	<70%	70–79%	≥80%	Total	p value
***Hospitals providing breast cancer surgery***					
Net gain	4 (5.6%)	14 (19.4%)	54 (75.0%)	72	<0.001^b^
Net loss	16 (19.8%)	31 (38.3%)	34 (42.0%)	81	
No significant net gain or loss	1 (7.8%)	1 (7.7%)	11 (84.6%)	13	
**Total**	21 (12.7%)	46 (27.7%)	99 (59.6%)	166	
***Hospitals providing colorectal cancer surgery***					
Net gain	5 (7.9%)	21 (33.3%)	37 (58.7%)	63	0.010^b^
Net loss	10 (15.2%)	34 (51.5%)	22 (33.3%)	66	
No significant net gain or loss	4 (11.8%)	8 (23.5%)	22 (64.7%)	34	
**Total**	19 (11.7%)	63 (38.7%)	81 (49.7%)	163	

a See methods for further explanation.

b Based on Fisher's exact tests.

### Potential spare surgical capacity

**Fig. 3:**
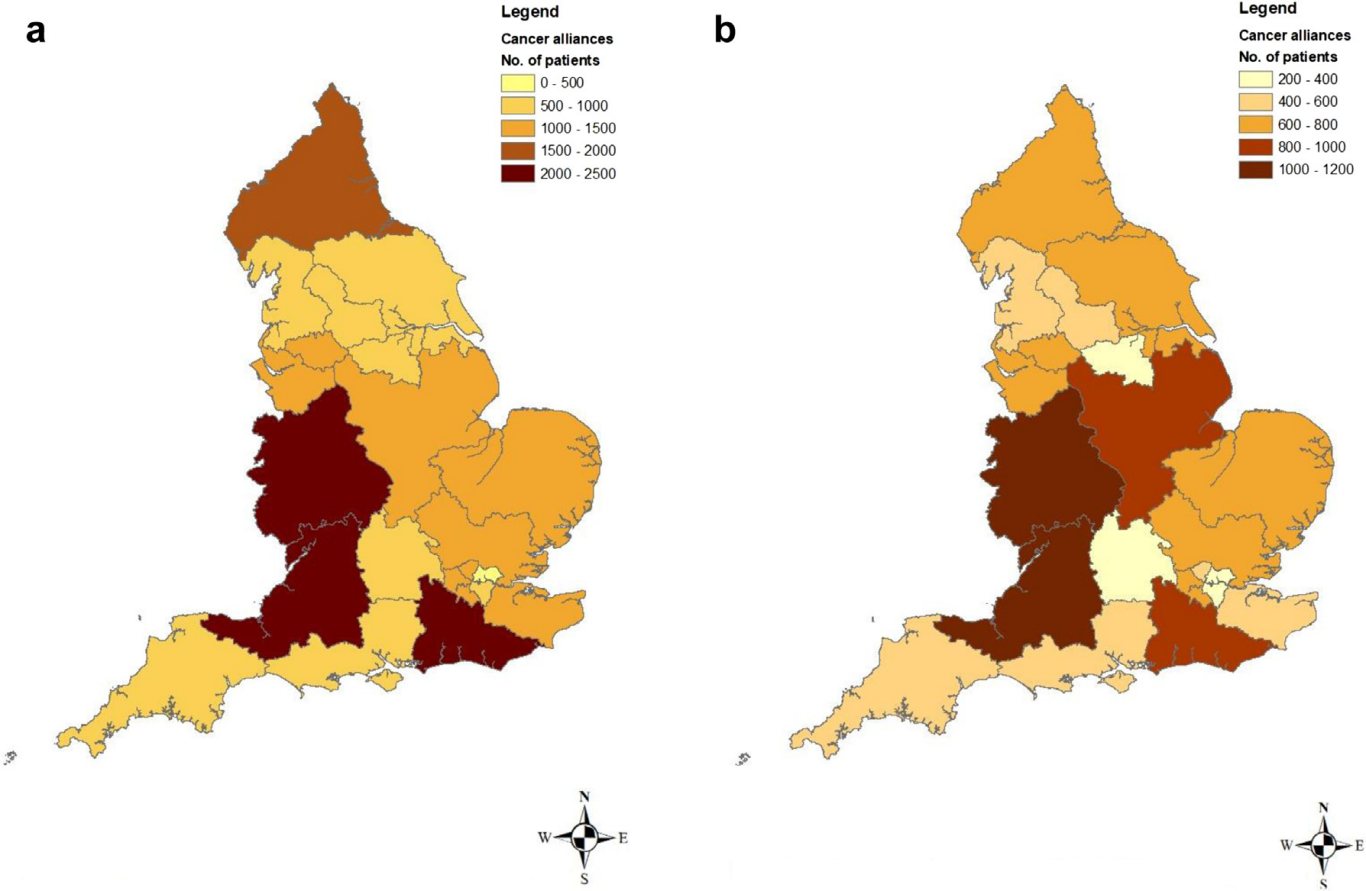
**a) Estimate of the spare surgical capacity between January 2016 and December 2018 for breast cancer surgical procedures across the 21 Cancer Alliances in England. b) Estimate of the spare surgical capacity between Jan 2016 and Dec 2018 for colorectal cancer surgery procedures across the 21 cancer alliances in England**.

Using our estimate of spare surgical capacity at national level, we projected that during the 3-year study period there was an annual spare capacity of 8389 breast cancer procedures (25.0% of the average surgical volume) and 4262 colorectal cancer procedures (25.9% of the average surgical volume). Fig. 3a and b demonstrate that the spare surgical capacity varied widely according to Cancer Alliance, with relatively high potential spare capacity seen both for breast cancer surgery and colorectal cancer surgery in the Somerset, Wiltshire, Avon and Gloucestershire Cancer Alliance and for breast cancer also in the Surrey and Sussex Cancer Alliance.

## Discussion

This study of the mobility of patients undergoing breast cancer surgery and colorectal cancer surgery in the English NHS demonstrates that some hospitals are performing more or fewer procedures than expected if all patients would be treated at their nearest hospitals. We found that hospitals with a net loss of patients had more potential spare capacity than hospitals with a net gain of patients. At the national level, we projected an additional spare capacity of about 8000 surgical procedures for breast cancer and about 4000 colorectal surgical procedures, which if fully used could increase the annual number of patients having breast cancer surgery and colorectal cancer surgery by 25%.

The management of the cancer backlog post COVID-19 pandemic is one of the UK's major political and clinical issues. Figures for September 2022 show for example that only 61.7% of patients are receiving treatment within 62 days of a referral, compared to 82.3% in the period between April 2017 and March 2018).^28^ As a result, patients are increasingly turning to the private health sector for treatment, and ongoing delays treatment pathways, especially surgical are likely to result in significant numbers of avoidable excess deaths.^29^

Our findings suggest that the English NHS does potentially have spare surgical capacity for two of the most common cancer pathways and therefore has several policy implications. First it is likely that these findings are not unique to the NHS and apply to many other countries with state-funded healthcare of universal health insurance coverage that allow patients to choose where they have their cancer treatment. Second, our results give an indication of the upper limit of the potential spare cancer surgery capacity if hospitals can perform continuously at their maximum level. We recommend translating this methodology across a range of cancer surgeries to quantify and establish regionally where surgical capacity is available to support service planning.

Third, NHS England, just as any national or regional health care commissioner, is currently modelling the gap between required capacity and actual capacity for cancer services as well as potential inefficiencies.^30^ Our study highlights the importance of considering patient mobility as a factor influencing cancer waiting times and available potential spare capacity in hospitals providing cancer surgery. It is important to note that so far, the policy debate about cancer waiting times focused on speeding up processes within hospitals^31^, ^32^, ^33^, ^34^, ^35^, ^36^, ^37^, ^38^ rather than on how patients are allocated between hospitals. However, we demonstrate that the variation between hospitals in actual surgical capacity usage (or conversely, in spare capacity) follows patient mobility patterns: hospitals that lose patients from their catchment area to other hospitals are typically those hospitals with more potential spare capacity.

Previous research including from our own study team has demonstrated that hospitals that are losing patients from their local area to other hospitals may be perceived to not offer the same level of quality of care or facilities as other hospitals, or do not have the same level of technical expertise or resources (e.g., critical care capacity) to perform specific procedures necessitating referral by the primary care or secondary care teams.^12^^,^^13^^,^^16^^,^^18^^,^^39^ These findings demonstrate the reasons for patient mobility are multifactorial. In our study we found that hospitals with a net loss of patients were less likely to offer specific specialist procedures such as breast reconstruction surgery and pelvic exenteration.

Fourth, a possible reason for the spare capacity in hospitals with a net loss of patients is that they are not using their available capacity efficiently, which could reflect the quality of internal management processes.^40^ We therefore would recommend an evaluation of hospitals with a net loss of patients to estimate the expected surgical capacity if all local patients were to receive treatments at these hospitals. Policies may also need to be considered which limit patient mobility (i.e., allowing patients to bypass their nearest hospital for treatment), only to those patients who may benefit from treatment in a hospital further away because of the technical complexity of the surgical procedure they require.

In England, a national long-term plan for the NHS, published in 2019, puts the 21 Cancer Alliances and the 42 Integrated Care Boards (the newly established regional organisations that will coordinate NHS care in England), at the heart of managing and coordinating cancer care pathways regionally.^8^^,^^41^^,^^42^ The regional bodies should be encouraged to facilitate service planning by mapping patient mobility and monitoring waiting times and ‘actual surgical capacity usage’ in the hospitals in their region.

To utilise surgical capacity efficiently and effectively the mobility of patients’ needs to be coordinated and allocated regionally based on the needs of individual patients. This would also take into account the resources and skill mix available at a given hospital to perform particular procedures based on complexity e.g., pelvic extenteration.^43^ This is not a straightforward task, and we therefore propose a managed care system using the specialist cancer multidisciplinary teams that have been established since 2000 to review the treatment plan for all newly diagnosed cancer patients within regionally collaborating NHS cancer care providers.^44^

A more radical consideration is that existing patient mobility leads to further centralisation of services and higher cancer treatment volumes at particular hospitals, which may be a consideration, especially for more complex surgical techniques, such as rectal cancer surgery.^45^ The potential spare capacity in hospitals losing cancer patients to other hospitals may be used for less complex procedures and elective treatments of benign conditions. Moves towards this type of regional coordination and centralisation of cancer services was evident during the COVID-19 pandemic.^46^ We have recently published a health services planning tool that can be used to understand the expected consequences that centralisation scenarios of cancer services has on travel burden, equity, and hospital capacity prior to implementation.^45^

Our study has several limitations. The study period does not include the COVID-19 pandemic. Therefore, we have no information to what extent the pandemic has changed the pre-existing patient mobility patterns. However, the number of hospitals performing surgery after the pandemic has remained stable and surgical activity has largely returned to pre-pandemic levels,^47^ although different types of procedures may have been adopted. In addition, the hospitals that we have identified as not meeting their waiting time targets continue to not to do so in 2021/2022.^48^ The method we have developed for estimating potential spare capacity can be applied as soon as surgical activity data covering the post-COVID-19 period becomes available.

We also acknowledge that a hospital's spare capacity is an elusive concept. Our estimates are based on a statistical approach that identifies fluctuations in surgical volumes over time within individual hospitals. This approach assumes that there are no systematic changes in the hospitals' actual capacity during the study period and that the case mix of the patients is stable. However, the model can be adapted to consider systematic changes in actual capacity over time as well as emergency operative caseloads.

We used a six-month moving average of the number of procedures performed in each hospital to estimate maximum surgical capacity. The use of moving averages over shorter periods, for example three months, would have led to higher maximum surgical capacity and in turn higher spare capacity estimates. We felt that six-month averages capture a level of capacity that a hospital can maintain over a sustained period. Peaks in capacity observed over shorter periods than six months are more likely to be a response to events that are not under the control of the hospital organisation. We do acknowledge that actual surgical capacity usage depends on many factors, including bed availability and workforce issues as well as competition from other cancer specialties for theatre space, that all need to be actively addressed and managed which may not be possible without additional investment.

We would also add that our definition of maximum surgical capacity depends on fluctuations in the volumes of surgical procedures over time. If hospitals consistently perform below their maximum capacity, this spare capacity would not be recognised by our approach.

It is important to note that the aim of our study was to describe how potential spare capacity is associated with patterns of patient mobility rather than to produce exact estimates of the spare capacity in each hospital. Further work, especially internal audits of the available work force and the actual usage of existing facilities in hospitals is needed to establish to what extent the potential spare capacity that we estimated using statistical criteria can be mobilised to tackle the cancer backlog. In this regard we support a regionally coordinated approach through the existing cancer MDTs structures.

A strength of the study is that we observe patterns of patient mobility over a 3-year period to ascertain net gains and losses. A detailed exploration of the reasons for the patterns of patient mobility is beyond the scope of this study but warrants more in-depth qualitative investigation within hospitals.

In summary we identified NHS hospitals in England that were performing fewer breast and colorectal cancer surgery procedures than expected if all eligible patients would have been treated at the hospital nearest to them. Hospitals that had a net loss of patients were estimated to have potentially more spare capacity than hospitals with a net loss. Our findings demonstrate the need for regional coordination to optimise the use of cancer surgery capacity and reduce cancer waiting times, using already existing regional coordinating structures. If all spare capacity as defined in our study could be used, there would be additional annual capacity for about 12,000 surgical procedures or 25% of the average volumes of breast and colorectal cancer procedures that are carried out.

## Contributors

AA and JvdM were involved in conceptualisation of this study. AA and LH and were involved in formal analysis. AA, LH, JvdM were involved in methodology. AA, RS, KH, VS, JvdM were involved in data interpretation. AA and LH wrote the original draft of the paper. LH produced the manuscript figures and tables. AA, LH, RS, KH, VS and JvdM were involved in reviewing and editing drafts of the paper.

## Data sharing statement

This study was based on English national cancer registry data. We do not own these data and hence are not permitted to share them in the original form. The data are available from the Office for Data Release at Public Health England. For access, please email odr@phe.gov.uk.

## Declaration of interests

We declare no competing interests.

## Appendix A Supplementary data

Caption for Supplementary Tables S1–S6 and Figs. S1–S3
