# Supplementary material for: Managing the cancer backlog: a national population-based study of patient mobility, waiting times and ‘spare capacity’ for cancer surgery
Source: Lancet Reg Health Eur. 2023 May 3;30:100642. doi: 10.1016/j.lanepe.2023.100642 (PMC10350851; doi:10.1016/j.lanepe.2023.100642)
Supplement: Caption for Supplementary Tables S1–S6 and Figs. S1–S3 [file mmc1.docx]

**Supplemental Data Captions**

**Appendix Figure 1**. Patient flow chart for breast cancer

**Appendix Figure 2**. Patient flow chart for colorectal cancer

**Appendix Table 1.** Characteristics of patients that received care at their nearest hospital (remainers) or at an alternative more distant hospital (leavers) for breast and colorectal cancer surgery.

**Appendix Table 2.** Characteristics of hospitals providing breast cancer surgery with a net gain or loss of patient due to patient mobility.

**Appendix Table 3.** Characteristics of hospitals providing breast cancer surgery with a net gain or loss of patient due to patient mobility.

**Appendix Table 4**. Number of hospitals performing both breast and colorectal cancer surgery that had a net gain or loss of patients for both procedure types.

**Appendix Table 5.** Proportion of hospital performing breast cancer surgery or colorectal cancer surgery with a net gain or loss of patients that met their waiting time target.

**Appendix Table 6**. Hospitals categorised according to their actual surgical capacity usage based on 6-month moving averages of surgical volumes for breast and colorectal cancer.

**Appendix Figure 3a**. Rate of hospital bypassing between Jan 2016 and Dec 2018 for breast cancer surgery procedures across cancer alliances in England.

**Appendix Figure 3b.** Rate of hospital bypassing between Jan 2016 and Dec 2018 for colorectal cancer surgery procedures across cancer alliances in England
