# Supplementary material for: Managing the cancer backlog: a national population-based study of patient mobility, waiting times and ‘spare capacity’ for cancer surgery
Source: Lancet Reg Health Eur. 2023 May 3;30:100642. doi: 10.1016/j.lanepe.2023.100642 (PMC10350851; doi:10.1016/j.lanepe.2023.100642)

**Supplemental Data**

**Appendix Figure 1**. Patient flow chart for breast cancer.

**Appendix Figure 2**. Patient flow chart for colorectal cancer.

**Appendix Table 1.** Characteristics of patients that received care at their nearest hospital (remainers) or at an alternative more distant hospital (leavers) for breast and colorectal cancer surgery.

|  | **Breast cancer (n=100,574)** | | | | | **Colorectal cancer (n=49,441)** | | | | |
| --- | --- | --- | --- | --- | --- | --- | --- | --- | --- | --- |
|  | **Leavers** | | **Remainers** | | ***p* value^a^** | **Leavers** | | **Remainers** | | ***p* value^a^** |
|  | **n** | **%** | **n** | **%** |  | **n** | **%** | **n** | **%** |  |
| **No. of patients** | 33,206 | 33.0 | 67,368 | 67.0 |  | 14,018 | 28.4 | 35,423 | 71.7 |  |
| **Age, mean (SD)** | 60.3 (12.7) | | 61.4 (13.1) | |  | 67.3 (12.1) | | 69.1 (11.5) | |  |
| 18-49 | 6,946 | 20.9 | 13,402 | 19.9 | <0.001 | 1,108 | 7.9 | 1,996 | 5.6 | <0.001 |
| 50-59 | 8,778 | 26.4 | 16,377 | 24.3 |  | 2,161 | 15.4 | 4,703 | 13.3 |  |
| 60-69 | 9,354 | 28.2 | 18,239 | 27.1 |  | 4,117 | 29.4 | 9,842 | 27.8 |  |
| 70+ | 8,128 | 24.5 | 19,350 | 28.7 |  | 6,632 | 47.3 | 18,882 | 53.3 |  |
| **IMD** |  |  |  |  |  |  |  |  |  |  |
| 1st quintile - least deprived | 7,665 | 23.1 | 15,049 | 22.3 | 0.001 | 3,599 | 25.7 | 7,836 | 22.1 | <0.001 |
| 2nd quintile | 7,497 | 22.6 | 15,727 | 23.3 |  | 3,310 | 23.6 | 8,424 | 23.8 |  |
| 3rd quintile | 6,983 | 21.0 | 14,095 | 20.9 |  | 2,905 | 20.7 | 7,561 | 21.3 |  |
| 4th quintile | 6,094 | 18.4 | 12,049 | 17.9 |  | 2,369 | 16.9 | 6,185 | 17.5 |  |
| 5th quintile - most deprived | 4,967 | 15.0 | 10,448 | 15.5 |  | 1,835 | 13.1 | 5,417 | 15.3 |  |
| **Rural** |  |  |  |  |  |  |  |  |  |  |
| Rural | 7,966 | 24.0 | 13,903 | 20.6 | <0.001 | 3,564 | 25.4 | 7,911 | 22.3 | <0.001 |
| Urban (non-London) | 19,612 | 59.1 | 47,494 | 70.5 |  | 8,177 | 58.3 | 24,750 | 69.9 |  |
| London | 5,628 | 17.0 | 5,971 | 8.9 |  | 2,277 | 16.2 | 2,762 | 7.8 |  |
| a. Based on Pearson’s chi-square test. |  |  |  |  |  |  |  |  |  |  |

**Appendix Table 2.** Characteristics of hospitals providing breast cancer surgery with a net gain or loss of patient due to patient mobility.

| **Breast Cancer** | **Net gain (n=72)** | **Net loss (n=81)** | **Total (n=153)** | ***p* value^a^** |
| --- | --- | --- | --- | --- |
|  |  |  |  |  |
| Comprehensive cancer centre | 26 (57.8%) | 19 (42.2%) | 45 | 0.09 |
| Non-comprehensive cancer centre | 46 (42.6%) | 62 (57.4%) | 108 |  |
|  |  |  |  |  |
| Breast reconstruction centre | 57 (57.0%) | 43 (43.0%) | 100 | **0.001** |
| Non- reconstruction centre | 15 (28.3%) | 38 (71.7%) | 53 |  |
|  |  |  |  |  |
| CQC rating - Outstanding/Good/Requires improvement | 67 (48.6%) | 71 (51.5%) | 138 | 0.26 |
| CQC rating - Inadequate | 5 (33.3%) | 10 (66.7%) | 15 |  |
|  |  |  |  |  |
| Research activity - quintiles 1-4 | 55 (44.0%) | 70 (56.0%) | 125 | 0.11 |
| Research activity - quintile 5 (highest) | 17 (60.7%) | 11 (39.3%) | 28 |  |
| a. Based on Pearson's chi-square test. | |  |  |  |

**Appendix Table 3.** Characteristics of hospitals providing breast cancer surgery with a net gain or loss of patient due to patient mobility.

| **Colorectal cancer** | **Net gain (n=63)** | **Net loss (n=66)** | **Total (n=129)** | ***p* value^a^** |
| --- | --- | --- | --- | --- |
|  |  |  |  |  |
| Comprehensive cancer centre | 19 (54.3%) | 16 (45.7%) | 35 | 0.45 |
| Non-comprehensive cancer centre | 44 (46.8%) | 50 (53.2%) | 94 |  |
|  |  |  |  |  |
| Robotic centre | 9 (64.3%) | 5 (35.7%) | 14 | 0.22 |
| Non-robotic centre | 54 (47.0%) | 61 (53.0%) | 115 |  |
|  |  |  |  |  |
| Specialist colorectal centre | 20 (62.5%) | 12 (37.5%) | 32 | 0.08 |
| Non-specialist colorectal centre | 43 (44.3%) | 54 (55.7%) | 97 |  |
|  |  |  |  |  |
| CQC rating - Outstanding/Good/Requires improvement | 58 (50.0%) | 58 (50.0%) | 116 | 0.43 |
| CQC rating - Inadequate | 5 (38.5%) | 8 (61.5%) | 13 |  |
|  |  |  |  |  |
| Research activity - quintiles 1-4 | 46 (46.5%) | 53 (53.5%) | 99 | 0.33 |
| Research activity - quintile 5 (highest) | 17 (56.7%) | 13 (43.3%) | 30 |  |
| a. Based on Pearson's chi-square test. |  |  |  |  |

**Appendix Table 4**. Number of hospitals performing both breast and colorectal cancer surgery that had a net gain or loss of patients for both procedure types.

|  | **Hospitals providing colorectal cancer surgery** | | |  |  |
| --- | --- | --- | --- | --- | --- |
|  | Net gain | Net loss | No significant difference | **Total** | ***p* value^a^** |
| **Hospitals providing breast cancer surgery** |  |  |  |  |  |
| Net gain | 35 (67.3%) | 7 (13.5%) | 10 (19.2%) | 52 | <0.001 |
| Net loss | 10 (15.9%) | 39 (61.9%) | 14 (22.2%) | 63 |  |
| No significant difference | 2 (20.0%) | 3 (30.0%) | 5 (50.0%) | 10 |  |
| **Total** | 47 (37.6%) | 49 (39.2%) | 29 (23.2%) | 125 |  |
| a. Based on Fisher's exact tests. | |  |  |  |  |

**Appendix Table 5.** Proportion of hospital performing breast cancer surgery or colorectal cancer surgery with a net gain or loss of patients that met their waiting time target.

|  | **Waiting time target**  **not met** | **Waiting time target**  **met** | **Total** | ***p* value** |
| --- | --- | --- | --- | --- |
| ***Hospitals providing breast cancer surgery*** | **Number (%)** | **Number (%)** | **Number** |  |
| Net gain of patients | 21 (29.2%) | 51 (70.8%) | 72 | 0.09^a^ |
| Net loss of patients | 12 (14.8%) | 69 (85.2%) | 81 |  |
| No significant difference | 3 (23.1%) | 10 (76.9%) | 13 |  |
| **Total** | 36 (21.7%) | 130 (78.3%) | 166 |  |
|  |  |  |  |  |
| ***Hospitals providing colorectal cancer surgery*** |  |  |  |  |
| Net gain of patients | 17 (27.0%) | 46 (73.0%) | 63 | 0.29^b^ |
| Net loss of patients | 12 (18.3%) | 54 (81.8%) | 66 |  |
| No significant difference | 5 (14.7%) | 29 (85.3%) | 34 |  |
| **Total** | 34 (20.9%) | 129 (79.1%) | 163 |  |
| a. Based on Fisher's exact tests. b. Based on Pearson’s chi-square test. | |  |  |  |

**Appendix Table 6**. Hospitals categorised according to their actual surgical capacity usage based on 6-month moving averages of surgical volumes for breast and colorectal cancer.

|  | **Number of hospitals** | **%** |
| --- | --- | --- |
| ***Hospitals providing breast cancer surgery*** |  |  |
| < 70% | 21 | 12.7 |
| 70 - 79.99% | 46 | 27.7 |
| >= 80% | 99 | 59.6 |
| **Total** | **166** | **100** |
|  |  |  |
| ***Hosptials providing colorectal cancer surgery*** |  |  |
| < 70% | 19 | 11.7 |
| 70 - 79.99% | 63 | 38.7 |
| >= 80% | 81 | 49.7 |
| **Total** | **163** | **100** |

**Appendix Figure 3a**. Rate of hospital bypassing between Jan 2016 and Dec 2018 for breast cancer surgery procedures across cancer alliances in England.


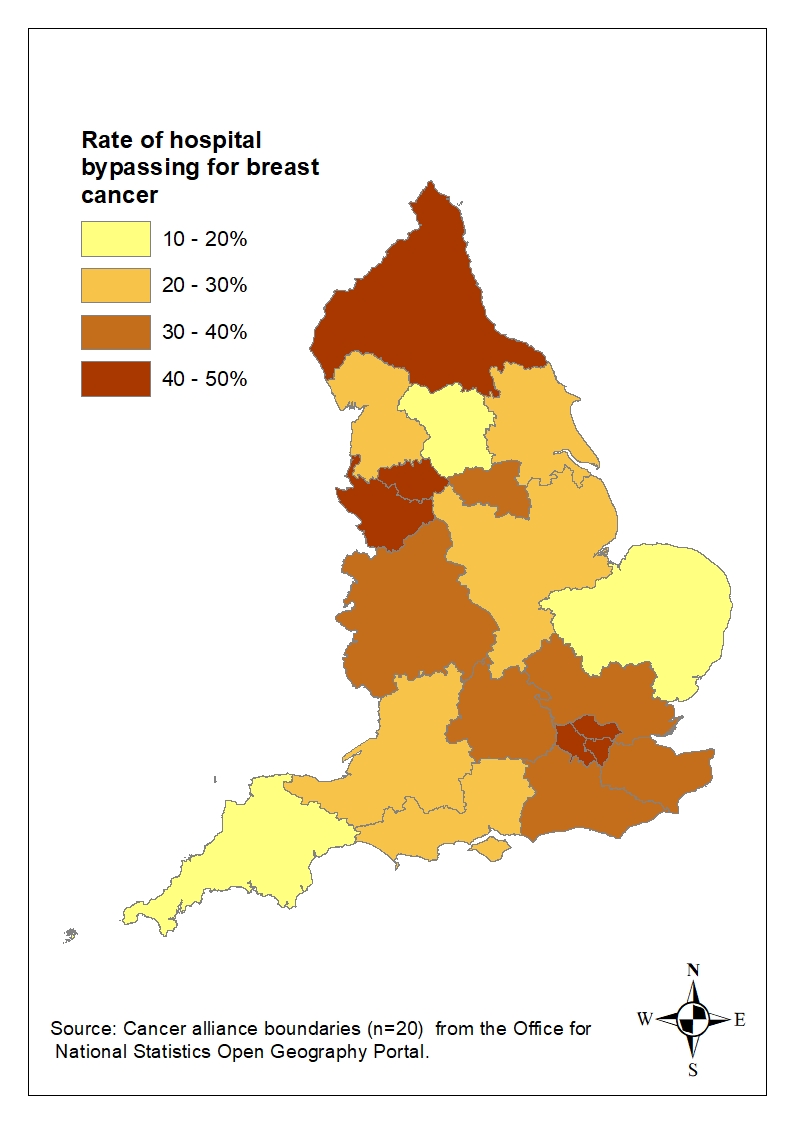


**Appendix Figure 3b**. Rate of hospital bypassing between Jan 2016 and Dec 2018 for colorectal cancer surgery procedures across cancer alliances in England.


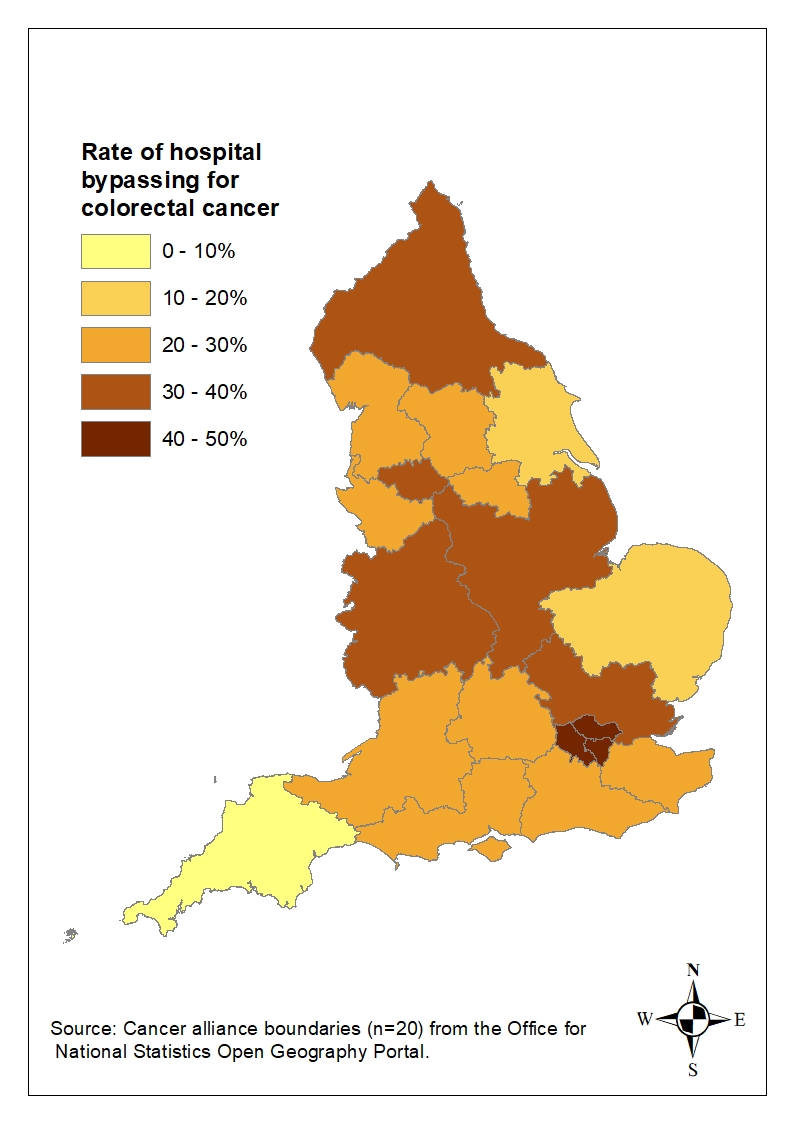

Supplement: Supplementary Tables S1–S6 and Figs. S1–S3 [file mmc2.docx]
